# An Integrated Review of Pesticides and Antibiotics in Agricultural Environments: Occurrence, Cross-Media Transport, and Plant Uptake

**DOI:** 10.3390/foods15081436

**Published:** 2026-04-20

**Authors:** Jie Li, Qing Yan, Bai Du, Guozhong Feng

**Affiliations:** State Key Laboratory of Rice Biology, China National Rice Research Institute, Chinese Academy of Agricultural Sciences, Hangzhou 311400, China; 821012410010@caas.cn (J.L.); ppk8848@163.com (B.D.)

**Keywords:** agroecosystems, environmental monitoring, soil–crop transfer, cross-media transport, emerging contaminants

## Abstract

With the continuing intensification of modern agriculture, pesticides and antibiotics are extensively used to control pests and diseases, but their improper use and indirect inputs have resulted in widespread contamination of agricultural environments and food products. This review synthesizes how these contaminants enter agroecosystems, their occurrence across soils, waters and agricultural products, and the processes that redistribute residues across air–water–soil interfaces and into the soil–plant continuum. We summarize cross-media transport pathways (e.g., runoff/leaching, volatilization–deposition and irrigation-driven redistribution) and relate environmental exposure to plant uptake using a harmonized indicator set, including the bioconcentration factor (BCF), translocation factor (TF), octanol–water partition coefficient (log Kow) and soil organic carbon–water partition coefficient (Koc). We further discuss key determinants of crop accumulation, including compound-specific properties, soil characteristics and plant physiological traits, and highlight how these factors jointly shape residue profiles in edible tissues. Finally, we outline research priorities for source reduction, standardized multi-matrix surveillance, fate-to-uptake modeling, and microbiome-enabled remediation strategies to support pollution control, food safety and public health.

## 1. Introduction

Pesticides and antibiotics are widely used in agricultural practice to control weeds, insect pests, and diseases to safeguard crop yields [1,2] and to maintain livestock health [3]. Compared with no control, the judicious application of pesticides can substantially increase crop yields: insecticides by approximately 36%, herbicides by about 50%, and fungicides by around 6% [4]. The use of antibiotics reduces the burden of livestock diseases and plays a major role in global animal production [5]. In addition, antibiotics such as streptomycin and oxytetracycline are important tools for suppressing bacterial epiphytotics in crops [6]. To meet rising food demand, global pesticide consumption has continued to increase; according to FAO, total pesticide use reached about 4.10 million tonnes in 2019, reflecting steady growth over time. For antibiotics, one study projects that global livestock antibiotic use (AMUQ) will increase by approximately 29.5% by 2040 relative to 2019 [7].

However, the excessive and improper use of pesticides and antibiotics in real-world agricultural production has resulted in continual inputs of diverse compounds into the environment and their subsequent transport, leading to widespread residues across agricultural ecosystems [8,9,10,11]. Recent studies have shown that approximately 64% of global agricultural land is at risk of pesticide pollution, with 31% classified as high-risk areas [12]. In addition, monitoring surveys of rivers worldwide have revealed that antibiotic concentrations at more than 25% of sampling sites exceed ecological risk thresholds [13].

Due to their high environmental persistence and bioaccumulation potential, these widely distributed contaminants can be absorbed and accumulated by crops [14], thereby entering the human body through the food chain and posing potential health risks. Studies have confirmed that vegetables, soybeans, and maize are capable of accumulating antibiotics from contaminated irrigation water [15,16], while crops such as cucumber and cowpea have been shown to take up and retain pesticide residues [17].

Accumulation of pesticides and antibiotics in crops introduces these chemicals into the food chain, creating an important human exposure route that poses potential health risks. Dietary exposure to pesticides has been associated with multiple adverse outcomes, including non-Hodgkin lymphoma, multiple myeloma, prostate cancer, Parkinson disease, as well as cognitive and respiratory disorders [18]. In recent years, exposure to low doses of antibiotics has been linked to numerous human health concerns, including obesity, carcinogenicity, reproductive effects, and teratogenicity [19], and residues in food have been implicated in these toxic effects [20,21]. Recent food-monitoring studies further indicate that pesticide residues remain frequently detectable in commonly consumed fruits and vegetables. A 17-year survey of 1223 apple samples detected 57 active substances in 64.2% of samples and identified compound-specific acute dietary risks for both children and adults, with children showing greater vulnerability; similarly, a large-scale study of 590 minor vegetable samples detected pesticide residues in 39.8% of samples and reported unacceptable acute risk in some child-consumption scenarios. Recent monitoring studies on commercially relevant fruits and vegetables further show that pesticide residues remain common in food matrices and may translate into differentiated dietary risks across consumer groups. Multi-year surveillance of apples [22] and a large-scale survey of minor vegetables [23] both reported frequent residue detection and identified higher concern in some child-exposure scenarios. In addition, contaminated foods can introduce antibiotic resistance genes (ARGs) into the human gastrointestinal tract [24], potentially undermining the effectiveness of antibiotics against common infections [25].

Beyond direct exposure concerns, this issue is also embedded in formal regulatory and governance systems. For pesticides, residue oversight is typically based on MRLs; Codex defines a pesticide MRL as the legally permitted maximum residue concentration in food or feed under good agricultural practice [26]. In the European Union, coordinated control programmes are implemented to verify compliance with MRLs and assess consumer exposure [27], whereas in the United States, analogous surveillance is conducted through the USDA Pesticide Data Program [28]. By contrast, the governance of antibiotic-related risks, particularly AMR, is increasingly framed within a One Health approach linking human, animal, plant, and environmental health [29]. Nevertheless, recent reviews suggest that integrated AMR surveillance remains insufficiently operationalized [30] and still requires stronger intersectoral coordination and governance capacity [31,32].

However, extant studies are still scattered, often concentrating on isolated aspects of the problem, such as contamination in a single environmental matrix, transport between compartments, or transfer from soil to plants, without integrating these elements into a comprehensive analysis. Consequently, there is a need for a clear framework that links environmental occurrences across various matrices with cross-media transport and the accumulation of residues in edible plant tissues. The development of standardized indicators and predictive models that connect environmental exposure to crop residues is limited, hindering effective monitoring and food-safety management.

Against this backdrop, this review summarizes the current status of pesticide and antibiotic contamination, examines environmental migration processes in plant–soil systems as well as the mechanisms of plant uptake and accumulation, and discusses the implications for food safety management and multi-matrix monitoring. This review aims to integrate occurrence, migration, and uptake into a unified analytical framework that links environmental distribution with the transfer of residues to edible crops. Finally, this paper outlines potential control strategies, including source reduction, microbiologically mediated remediation, and regional monitoring, with the aim of providing a more systematic scientific basis for contaminant management and food safety protection in agricultural environments.

To facilitate readers in reproducing the literature screening process described in this review, the detailed search strategy, inclusion criteria, and screening procedures are presented in Figure S1.

## 2. Pollution Status of Pesticides and Antibiotics

### 2.1. Common Contaminant Types and Sources

Pesticides are widely applied for the control of crop pests and diseases, while antibiotics are frequently used in livestock production and, to a lesser extent, in plant disease management. Based on their intended use, pesticides can be categorized into insecticides, fungicides, and herbicides [6]. Representative insecticide groups include organophosphates (e.g., chlorpyrifos, malathion), pyrethroids (e.g., cypermethrin), and neonicotinoids (e.g., imidacloprid), which are primarily used for insect control. Fungicides such as triazoles (e.g., tebuconazole) and benzimidazoles (e.g., carbendazim) target fungal pathogens, whereas herbicides like triazines (e.g., atrazine) and acetanilides (e.g., acetochlor) are widely applied to suppress weed growth in fields. Pesticides can also be classified according to their chemical structure [6], with major examples including organophosphates, organochlorines, carbamates, pyrethroids, neonicotinoids, and triazoles. Among them, organochlorine pesticides (e.g., DDT) have garnered global attention due to their environmental persistence and potential for biomagnification [33].

**Figure 1:**
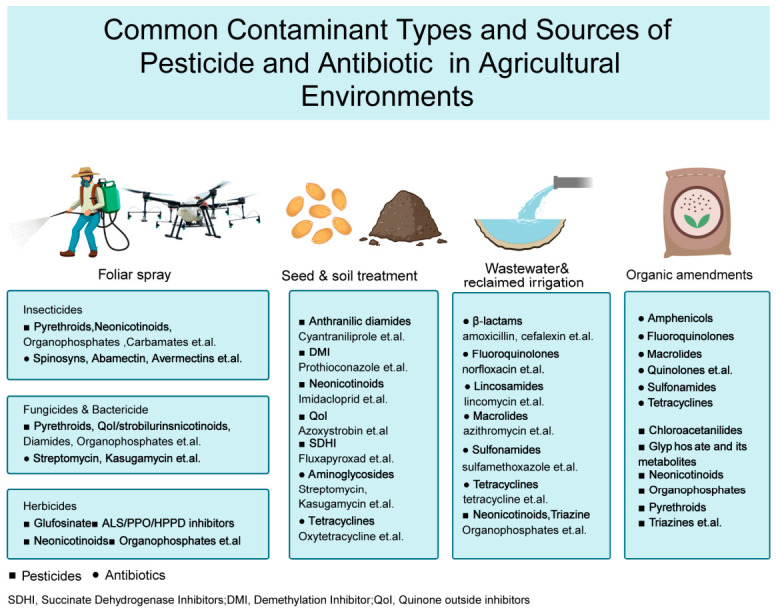
Common Contaminant Types and Sources of Pesticide and Antibiotic in Agricultural Environments.

In terms of antibiotics, commonly used classes in agricultural production include tetracyclines, sulfonamides, fluoroquinolones, macrolides, aminoglycosides, and lincosamides [34]. For instance, oxytetracycline (a tetracycline) and sulfamethoxazole (a sulfonamide) are widely employed for disease prevention in livestock and poultry [35], and can persist in the environment long after excretion. Fluoroquinolones such as enrofloxacin and ciprofloxacin are extensively used in both livestock and aquaculture due to their broad-spectrum activity, yet they exhibit high ecological toxicity and poor degradability [36]. Aminoglycosides like streptomycin and kasugamycin are among the few antibiotics officially approved for use in plant disease management [37]. Antibiotics can enter soil and aquatic environments through direct application, animal excreta, or irrigation with contaminated wastewater, leading to the accumulation of multiple antimicrobial resistance risks in the environment. Figure 1 summarizes the common contaminant types and major input sources of pesticides and antibiotics in agricultural environments.

The primary source of pesticide contamination is the direct application during crop cultivation, including foliar spraying [38], seed treatment [39], and other field practices. These applications lead to the accumulation of pesticide residues in soils, water bodies, and on or within crop tissues. In contrast, antibiotics typically enter the environment through indirect pathways. They are extensively used in livestock and poultry farming, but are often not fully metabolized within the animals. As a result, antibiotics are excreted in their parent or partially active forms and subsequently applied to agricultural fields through manure or organic fertilizer derived from animal waste [40]. This practice represents a major environmental dissemination route for both antibiotics and antibiotic resistance genes. In contemporary crop protection, direct application is the principal route by which pesticides enter agroecosystems. The most common practice is foliar spraying, which underpins the management of major diseases, insect pests, and weeds across staple and cash crops. Multiple studies have reported that appropriately designed spray programs can deliver effective field control of targets such as rice neck blast, cotton thrips, and lepidopteran pests of maize [41,42,43,44].

Soil application is likewise widely employed to manage subterranean insect pests, soil-borne diseases, and weeds, including pre-emergence control. Representative practices include soil incorporation of pesticides and in-furrow or band application to improve within-soil distribution and reduce phytotoxicity; the use of blended fumigants to suppress soil-borne pathogens; and pre-emergence herbicide treatments to lower early-season weed pressure [45,46,47].

Seed treatment is a common strategy within integrated pest management (IPM), conferring prophylactic protection before sowing to reduce disease and pest risks during the seedling stage; for example, SDHI-class treatments are used to manage seedling diseases in cereals and oilseed crops, anthranilic diamides (e.g., chlorantraniliprole) are employed for early-season insect control in maize, and benzimidazoles are applied to target specific seedling diseases in rice [48,49,50,51,52].

Antibiotics enter agricultural environments through three main pathways: (1) direct agricultural application for crop protection, (2) indirect introduction through reclaimed-water irrigation and the land application of sludge or biosolids, and (3) veterinary use followed by manure application.

(1) Crop protection is a critical pathway for antibiotic entry into agriculture. In some systems, antibiotics are deliberately administered to manage plant diseases. Representative examples include streptomycin for fire blight in apples, jinggangmycin for rice sheath blight, and tetracyclines for citrus huanglongbing [53,54,55,56,57,58,59]. (2) Following crop protection, another significant pathway is the reuse of wastewater, sludge, and reclaimed water. A wide variety of antibiotic classes have been identified in hospital, pharmaceutical, and municipal wastewater streams. The reintroduction of these contaminants into agroecosystems can occur through the use of reclaimed water for irrigation and the land application of sludge or biosolids. Incomplete removal of antibiotics during treatment results in variable effluent concentrations [60,61,62,63,64,65,66,67,68,69]. (3) Veterinary use and manure amendment represent another major pathway for antibiotics entering agricultural soils. Veterinary use of antibiotics in livestock production is substantial, and incomplete metabolism leads to residues in manure. Consequently, the application of manure and slurry to farmland represents a significant pathway by which antibiotics enter agricultural soils [7,70,71,72,73,74,75,76].

These pathways, which refer to the routes through which contaminants enter agricultural systems, are not always discrete in practice, because agricultural inputs themselves may already carry contaminant residues. A study of 15 bio-based fertilisers found pesticide or pharmaceutical compounds in most samples, with concentrations ranging from 4.1 to 181 μg/kg [77]. This finding indicates that agricultural amendments may inadvertently carry pesticides, antibiotics, and other contaminants, thereby contributing a hidden and persistent pollutant load to soils. Notably, the reported concentrations (4.1–181 μg/kg) approach levels shown in other studies to affect soil microbial communities and facilitate bioaccumulation. Furthermore, in certain regions, wastewater irrigation [78] and the reuse of treated effluent [79] remain important supplements to agricultural water supplies. In instances where treatment is deemed incomplete, municipal or livestock-derived wastewaters have the potential to introduce a mixture of pesticide and antibiotic residues into farmland systems, thereby establishing a persistent source of contamination.

### 2.2. Pollution Status in Soils and Water Bodies

Agricultural soils and irrigation-related water bodies serve as the primary environmental sinks for pesticides and antibiotics. Due to their persistence (long environmental half-lives) and strong sorption to soils (high Koc), these contaminants remain in agroecosystems over extend periods. To facilitate comparison, the key physicochemical properties and policy restrictions of the selected compounds are summarized in Tables S1 and S2.

#### 2.2.1. Pesticide Residues in Soils and Water Bodies

Driven by the rapid intensification of modern agriculture, global pesticide consumption has continued to rise in efforts to secure crop yields. In practice, overuse and misuse are common, leaving agricultural soils widely contaminated with residues of diverse pesticides at varying concentrations. As a national-scale example, a survey of 154 surface soil samples across China revealed that more than 76% of the samples contained 21–30 pesticide residues, indicating the widespread and complex nature of pesticide contamination in Chinese soils [80]. In long-term monitoring of wheat fields near Beijing, commonly detected compounds included carbendazim and imidacloprid, with tri fungicides (e.g., hexaconazole) occasionally reaching >1000 μg/kg (dry weight) [81]. Legacy organochlorines remain detectable in specific settings such as karst soils in Hubei (organochlorine pesticides, OCPs 0.16–43.10 μg/kg; dominated by DDTs and mirex) [82]. Across Europe, large-scale surveys reported pesticide residues in 97% of agricultural soils, with ≥2 substances in 88% of samples and a substantial fraction of high-risk fields under conventional management [83,84,85].

Beyond soils, pesticide residues are widely detected in surface and groundwater associated with agricultural activities such as irrigation and aquaculture. These residues comprise diverse compounds, with concentrations varying across a broad range.

Irrigation canals and agricultural catchments typically exhibit ng/L to low-μg/L levels of total pesticide concentrations, with multi-compound detections common. Across 64 monitored water bodies spanning 10 European countries and Argentina, total pesticide concentrations ranged 6.89–5860 ng/L [86]. By comparison, a basin-scale survey in Spain detected 35 actives, with bentazone peaking at 18,000 ng/L [87]. Near-coastal systems show broader chemical coverage: a Chinese aquaculture area reported up to 296 pesticide compounds with a small subset driving ecological risk [88], and the eastern Shandong coast documented concurrent legacy OCPs in seawater, sediments, and biota [89]. In the Beijing region, 10 pesticide compounds were identified in rivers, with concentrations from below detection to 323.44 ng/L [90]. Following direct application in agricultural fields, a substantial portion of pesticides can adsorb onto soil particles or migrate through leaching and surface runoff during rainfall or irrigation events, thereby contaminating surface and groundwater systems [91].

Shallow and hydrogeologically vulnerable aquifers are especially susceptible to pesticide contamination, with residues often detected at ng/L levels in agricultural settings. In a representative irrigated agricultural region of the North China Plain, groundwater contamination is dominated by atrazine and its transformation products desethylatrazine (DEA) and desisopropylatrazine (DIA), with high detection frequencies and pronounced spatial heterogeneity [92]. In southern Netherlands farmlands, transformation products such as BAM (2,6-dichlorobenzamide), DMS (N, N-dimethylsulfamide), and desphenyl-chloridazon were persistently and widely detected from 1980 to 2020, indicating high mobility and environmental persistence in groundwater [93]. Regarding legacy OCPs, shallow groundwater in Shouguang, Shandong Province, commonly contained OCP residues, with most samples below drinking-water standards but localized hotspots of elevated concentrations [94]; across multiple regions of India, OCPs were widespread in groundwater and frequently exceeded standards, reaching up to 890 ng/L in some areas, underscoring persistence, regional disparities, and strong links to contaminated soils [95]. By comparison, currently used pesticides such as neonicotinoids and fipronil-class compounds are widely detected in groundwater. In Minnesota (USA), clothianidin occurred in 41% of springs at up to 200 ng/L, with contamination concentrated in shallow (<40 m), oxic aquifers and spatial patterns strongly modulated by geology, land use, and groundwater age [96]. In agricultural areas of Korea, 26 pesticides were detected in shallow groundwater; metolachlor showed the highest detection frequency (10.13%) with a mean concentration of 12.30 ng/L. Detection frequencies in agricultural settings were significantly higher than in background areas, indicating widespread ng/L-level contamination and pronounced regional heterogeneity in shallow aquifer systems [97].

Overall, the available evidence indicates that both legacy and currently used pesticides are widely present in agricultural environmental compartments, particularly in soils and groundwater, although their detection frequencies and concentration ranges vary substantially among compounds and regions. Such variation likely reflects differences in historical usage, ongoing inputs, and local hydrogeological settings.

#### 2.2.2. Antibiotic Residues in Soils and Water Bodies

Antibiotics primarily enter agricultural environments through the application of livestock manure, sludge-based fertilizers, and irrigation with reclaimed water. While these materials are commonly regarded as valuable sources of nutrients in agriculture, they also represent significant sources of antibiotic contamination.

Antibiotics are widespread across agricultural environments, yet their distributions exhibit pronounced spatial heterogeneity. Concentrations and forms of occurrence (speciation and phase partitioning) are governed by multiple factors, including source inputs, intrinsic physicochemical properties, characteristics of the receiving media, and agricultural management practices.

Soils are the principal sink for antibiotics in agricultural environments, especially in fields amended with livestock manure. Evidence from across the globe indicates that agricultural soils are contaminated to varying degrees, with tetracyclines, sulfonamides, fluoroquinolones, and macrolides most frequently detected [98]. For example, in multiple vegetable soils across the Pearl River Delta (South China), tetracyclines (TCs) were widespread: more than 85% of samples contained three or more TCs, with a mean concentration of 78.05 μg/kg [99]. Tetracyclines and fluoroquinolones are largely concentrated in surface horizons and decline with depth, whereas sulfonamides often show the opposite pattern [100,101].

A survey of 24 representative antibiotics in Hong Kong rivers and coastal waters showed ubiquitous occurrence of tetracyclines (TCs; detection frequency 100%), while sulfonamides (SAs), fluoroquinolones (FQs; 78.60–100%), roxithromycin (RTM; 50%), and novobiocin (NOV; 50%) each exhibited detection frequencies ≥50%. Compared with river water, concentrations of most antibiotics were lower in seawater [102]. A survey in Xinjiang, China, reported total antibiotic concentrations of 17.37–84.09 ng/L in surface waters and 16.38–277.41 ng/L in groundwater. Norfloxacin was the most abundant compound in surface waters, whereas pefloxacin predominated in groundwater. These contrasting distributions were attributed to differences in antibiotic physicochemical properties and soil characteristics [103].

Residual antibiotics impose sustained selective pressure in the environment, promoting the selection and enrichment of antibiotic-resistant bacteria (ARB) carrying ARGs [104]. Importantly, ARG persistence does not necessarily decline in parallel with measurable antibiotic residues, because co-selection by non-antibiotic stressors and the genetic linkage of ARGs on mobile elements can maintain resistance even when direct antibiotic selection is weak or absent [105]. Accordingly, agricultural environments should be viewed not only as sinks of antibiotic residues, but also as reservoirs in which ARGs may be maintained and disseminated through mobile genetic elements and horizontal gene transfer [106].

Accordingly, a systematic synthesis of the occurrence and distribution of antibiotics and ARGs in soils and waters (surface water, groundwater, and irrigation water) is essential to delineate the full scope of antibiotic pollution in agroecosystems.

ARGs are now globally pervasive [72,107]. Accordingly, several studies have interrogated the global biogeography of ARGs to discern macro-scale regularities in their occurrence and distribution. A survey of 226 activated-sludge samples from 142 wastewater treatment plants (WWTPs) across six continents identified 179 ARGs spanning 15 antibiotic classes and defined 20 “core ARGs” detected in all samples, together accounting for 83.80% of total ARG abundance; β-lactam, glycopeptide, and tetracycline classes predominated [108]. While total ARG abundance did not differ significantly among continents, diversity was higher in Asia and community composition showed pronounced intercontinental differentiation. ARG abundance correlated positively with mobile genetic elements (MGEs), and more than half of the 1112 high-quality genomes recovered harbored mobilizable ARGs, underscoring WWTP activated sludge as a critical global reservoir and exchange hub for environmental ARGs [108]. Another study focused on the distribution of soil ARGs. Drawing on 1088 soil metagenomes worldwide, it identified 558 ARG subtypes across 23 classes, with a mean abundance of 121 ppm; abundances were significantly higher in agricultural than in non-agricultural habitats. A global map of ARG abundance revealed hotspots in East and South Asia, Western Europe, and the eastern United States, with elevated levels also observed at high latitudes, including New Zealand and Northern Europe [109].

Overall, the available evidence points to widespread but spatially heterogeneous occurrence of antibiotics and ARGs, shaped by anthropogenic inputs, host microbial communities, and mobile genetic elements. These patterns suggest that agricultural environments can serve not only as contaminant sinks, but also as reservoirs for resistance dissemination. Cross-study comparisons should nevertheless be interpreted cautiously, because reported occurrence patterns are strongly influenced by sampling design, analytical coverage, extraction efficiency, matrix effects, and method detection limits. Moreover, many monitoring studies focus primarily on parent compounds, whereas transformation products and other derived residues are much less consistently captured. Such methodological differences may partly contribute to the substantial variability in reported detection frequencies and concentration ranges across regions and studies.

#### 2.2.3. Co-Occurrence and Mixture

Pesticide and antibiotic residues are ecotoxic environmental pollutants with implications for human health; their pervasive co-occurrence undoubtedly poses a major challenge to the ecological integrity of agroecosystems and to food safety.

Moreover, pesticides and antibiotics frequently co-occur in real agricultural environments, resulting in complex patterns of co-contamination. For example, in China’s Haihe River System (HRS)—a representative agricultural irrigation region—researchers employed high-resolution mass spectrometry to screen 268 contaminants and identified 62 priority compounds for spatiotemporal tracking, encompassing both pesticides and antibiotics [110].

In midstream Ganges water samples, a total of 51 emerging organic contaminants (EOCs) and their metabolites were concurrently detected, including pharmaceuticals such as sulfamethoxazole (detection frequency 73%, maximum 63 ng/L) and pesticides such as diuron (detection frequency 100%, maximum 95 ng/L) and atrazine (detection frequency 82%, maximum 12 ng/L) [111]. These riverine examples underline a seasonal inversion: pharmaceutical-dominant mixtures during high-flow periods and pesticide-dominant mixtures in dry seasons. Notably, in a set of samples from the Niyang River on the Qinghai–Tibet Plateau, 33 antibiotics and six pesticides from the triazine and benzimidazole classes were co-detected; representative concentrations included erythromycin (median 5.20 ng/L) among antibiotics and carbendazim (median 66 ng/L) and thiabendazole (median 88 ng/L) among pesticides. Antibiotics predominated during the high-flow season, whereas pesticides predominated during the dry season [112].

Co-occurrence of pesticides and antibiotics is also evident in groundwater. In a typical irrigated agricultural region, four antibiotics (mean detection frequencies 37–100%) and five pesticides (27.50–88.30%) were simultaneously detected, with the highest summed concentration reaching 538 ng/L [92]. In a survey across the North China region, co-occurrence was widespread in farmland soils: pesticides ranged from non-detect to 3800 μg kg^−1^ and antibiotics from non-detect to 951 μg kg^−1^ across 530 samples [113]. In subsurface and soils, co-detection persists but is filtered by hydrogeology and sorption, yielding mixtures with fewer classes yet longer residence.

These co-occurrence patterns indicate that ecological risk in agricultural waters cannot be inferred solely from single-compound occurrence data [114,115]. In mixture toxicology, concentration addition (CA) and independent action (IA) are commonly used as reference models for estimating combined effects [114,116]. CA is generally applied as the default or conservative baseline for chemicals with similar or insufficiently resolved modes of action, whereas IA is more appropriate when components act through dissimilar pathways or target sites [116,117]. However, environmentally realistic mixtures do not always conform neatly to these idealized assumptions, and deviations such as synergistic or antagonistic effects may occur depending on mixture composition, concentration range, and biological endpoint [118,119]. Therefore, co-occurrence data are more appropriately interpreted in combination with mixture-oriented risk screening rather than on a compound-by-compound basis alone [115,120].

## 3. Environmental Migration of Pesticides and Antibiotics

### 3.1. Environmental Migration of Pesticides

**Figure 2:**
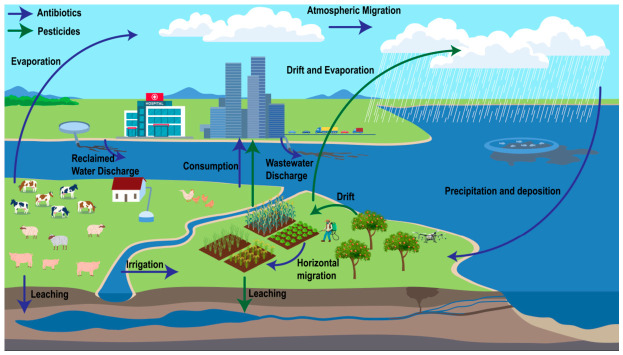
Cross-media transport pathways of pesticides and antibiotics in agroecosystems. This schematic summarizes the major sources and environmental processes governing the fate of pesticides (green arrows) and antibiotics (blue arrows) across the air–water–soil–crop continuum. Key pathways include inputs via wastewater/reclaimed-water discharge and irrigation, spray drift and volatilization during field application, long-range atmospheric transport followed by precipitation-driven deposition, surface runoff and horizontal migration, and downward leaching to deeper soil layers and groundwater.

Following direct application in agricultural settings, pesticides are not only retained within environmental matrices such as soil but are also transported beyond target areas via air masses and hydrological flows. As shown in Figure 2, pesticides and antibiotics can migrate across soil, water, air, and crop compartments through multiple transport pathways. When pesticides are applied using sprayers or unmanned aerial vehicles, a fraction fails to deposit on the intended crop and is carried by wind to non-target areas; this process is termed spray drift [121,122]. Drift from aerial application is substantially greater than that from ground-based spraying [123,124]. Pesticides that have drifted into surrounding environments may further volatilize or adsorb to particulate matter and be entrained by wind erosion into the atmosphere, thereby facilitating transport to even more distant non-target regions [124,125]. Such airborne pesticides have been detected not only over agricultural landscapes but also in remote mountain regions, coastal zones, and even the Arctic; notably, concentrations of atrazine in agricultural areas and in the Arctic differ by less than one order of magnitude, indicating long-range atmospheric transport, recirculation, and persistence in distant environments [126].

In contrast to legacy OCPs, which are mainly detected as persistent residues from historical use, many pesticides still used in modern agriculture continue to enter the atmosphere through ongoing application and are subsequently returned to crop and soil surfaces by dry and wet deposition. A survey from Turkey showed that pesticides in air deposit either directly via dry deposition—where benomyl exhibited the highest dry-deposition velocity—or via precipitation, with the highest concentrations in rainfall reported for benomyl and pymetrozine ranging from 4–31.73 ng/L [127]. In a separate monitoring campaign across three land-use categories in São Paulo State, Brazil, pesticides were detected in all rainwater samples (0.10–596 ng/L). In predominantly agricultural areas, both the detection frequency of 19 compounds and the total wet-deposition flux were significantly higher than in the metropolitan zone, indicating that rainfall is an important pathway for returning atmospheric pesticide residues to environmental surfaces, especially in agricultural settings [128].

After deposition, these residues may follow several major environmental fates. First, they may be adsorbed and retained in soils. For example, neonicotinoids such as imidacloprid and thiamethoxam are readily adsorbed by soils, with the process largely governed by clay minerals and associated with a risk of partly irreversible retention [129]. Higher soil organic matter also strengthens atrazine sorption by promoting hydrogen bonding and hydrophobic interactions [130]. Second, deposited residues may be washed into the surface mixed layer during rainfall or irrigation and subsequently leach into shallow groundwater [96,131,132]. Third, they may be exported in dissolved or particulate forms via runoff and erosion to field ditches and fluvial networks, while excessive irrigation can further accelerate their downward migration into subsurface aquifers [133]. A smaller fraction may also re-enter the atmosphere through re-volatilization or resuspension. Overall, these processes show that soils act as a major reservoir and redistribution hub for pesticide residues after application.

### 3.2. Environmental Migration of Antibiotics

The transport of antibiotics in the environment is a complex, multi-scale, and multi-medium process. Although antibiotics share many physicochemical transport pathways with other pesticides, including sorption, runoff, leaching, and deposition, their environmental migration also involves biologically mediated processes associated with ARGs, MGEs, and microbial hosts.

After release into agroecosystems, antibiotics partition among soil [134,135,136], water and air compartments, but are typically retained in soils and surface waters as long-term sinks. Key redistribution pathways include sorption–desorption, downward movement through the soil profile, and hydrologic transport via runoff and groundwater [137,138], with episodic atmospheric transport and deposition also reported [139,140,141]. In paddy red soils, colloidal mineral/organic particles (e.g., clay minerals, iron oxides and humic substances) can facilitate vertical and interfacial transport by forming reversible complexes with antibiotics and by modifying local adsorption capacity and microbial habitats [142].

ARGs are increasingly recognized as important environmental contaminants whose risks may, in some contexts, exceed those of the antibiotics themselves, because they can be exchanged among microorganisms and thereby promote the spread and persistence of resistance in environmental reservoirs [143]. The core mechanism of this dissemination is horizontal gene transfer (HGT)—that is, bacteria acquire genetic material from the surrounding environment via conjugation, transformation, or transduction rather than solely through vertical inheritance [144,145]. During HGT, MGEs—including plasmids, transposons, and integrons—serve as vectors or “shuttles” for ARGs, markedly accelerating their dissemination across bacterial species and lineages [146]. Consequently, elucidating the transport and fate of ARGs and MGEs across complex environmental matrices is crucial for assessing and managing ecological risks in agricultural systems, safeguarding food safety, and protecting human health.

Repeated, long-term application of animal manures laden with high concentrations of antibiotic residues, ARB, and ARGs markedly promotes the vertical migration of ARGs and MGEs within agricultural soils [147,148,149]. For example, ARGs have been detected throughout manure-amended soil profiles from 0 to 100 cm depth, demonstrating their movement into deeper horizons [150]. Soil properties are key regulators of this process: structural equation modeling (SEM) indicates that soil pH, organic carbon content, and soil texture, particularly sand fraction, are primary drivers of ARG vertical transport [151]. Beyond abiotic controls, the bacterial community itself is considered a principal determinant; certain ARGs are conveyed to deeper layers by their host bacteria rather than being governed solely by physicochemical factors [150]. Rainfall events significantly enhance ARG vertical migration and leaching [151], heightening the risk of groundwater contamination, while irrigation can mobilize ARGs, MGEs, and related contaminants in overland runoff, facilitating pollution of rivers, lakes, and other surface waters. Notably, wastewater discharged from hospitals and pharmaceutical plants carries ARGs that can disperse directly into surrounding aquatic environments via exchange within surface waters and through subsurface groundwater pathways [152,153]. ARB and their associated ARGs can be aerosolized, and airborne ARB/ARGs have been persistently detected in locally collected air samples across diverse settings—including wastewater treatment plants, composting facilities, intensive livestock operations, and industrial, urban, and rural environments [154,155,156,157,158]. Suspension of ARGs in the atmosphere is of particular concern because it can facilitate long-range transport and promote dissemination across environmental compartments and ecosystems. For example, a four-year monitoring campaign at a high-mountain observatory in the free troposphere analyzed rain and snow by qPCR and 16S rRNA gene sequencing, demonstrating intercontinental-scale atmospheric transport and deposition of ARGs and MGEs [159].

The environmental dissemination of ARGs is shaped by multiple factors. Integrating global soil metagenomes, a study employing structural equation modeling with 169 spatial covariates coupled with random forest prediction identified anthropogenic activities (livestock production, manure or reclaimed-water irrigation, and pesticide application) as the dominant drivers of ARG enrichment, whereas climate and soil nutrients act indirectly by modulating host communities [109]. Studies have shown that the spatial distribution of ARGs is influenced by climatic factors such as precipitation and temperature, while MGEs and heavy metals are the primary driving forces for their dissemination, contributing approximately 19% and 29%, respectively. These factors can facilitate the horizontal transfer of ARGs through co-selection mechanisms [160].

Taken together, pesticides and antibiotics are redistributed repeatedly among air, water, and soils, with soils acting as a major reservoir as well as a secondary source. Their environmental movement is driven by direct inputs, atmospheric deposition, leaching, runoff, erosion, and, in some cases, re-volatilization. For antibiotics, dissemination is further shaped by biological carriers and genetic elements, including ARB, ARGs, and MGEs, which can facilitate persistence and spread across environmental compartments. These coupled physicochemical and biological processes determine contaminant availability at the plant–air and plant–soil interfaces. Therefore, it is important to investigate how pesticides and antibiotics cross these interfaces, enter above- and below-ground tissues, and are transported within plants.

## 4. Soil-Mediated Plant Uptake, In-Plant Translocation and Metabolism

**Figure 3:**
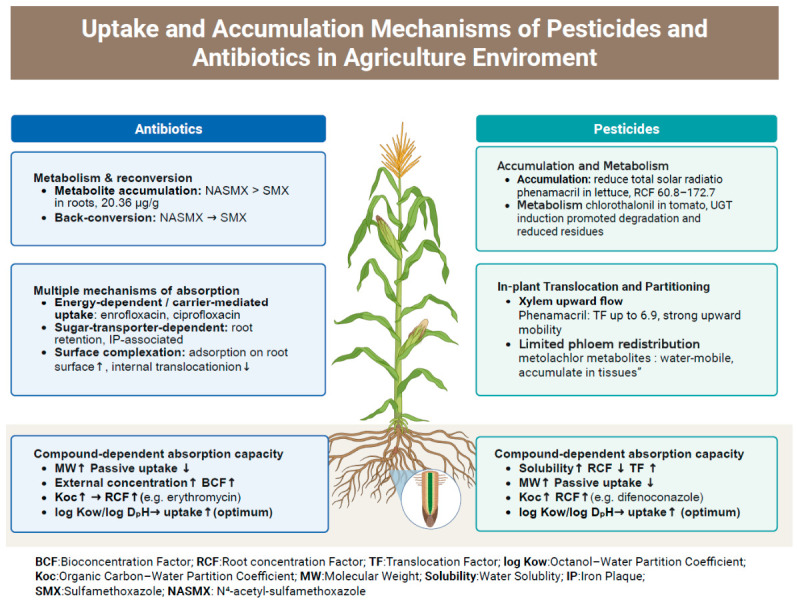
Uptake and Accumulation Mechanisms of Pesticides and Antibiotics in Agriculture Enviroment. Upward and downward arrows indicate an increase or decrease in the corresponding parameter or process, whereas rightward arrows indicate a promoting or causal relationship between factors (e.g., higher Koc leading to higher RCF).

In agroecosystems, xenobiotics can reach edible tissues through both belowground and aboveground pathways. Soil-mediated exposure represents the dominant and best-characterized pathway in many agricultural settings. For soil-mediated exposure, the process generally involves three coupled steps: (i) vertical transport in soil, (ii) root uptake, and (iii) in-plant translocation and storage. Direct uptake through leaves and stems may also occur after deposition or surface exposure. For consistency, we refer to key physicochemical descriptors (log Kow, water solubility, Koc, molecular weight) and use standard metrics: the bioconcentration factor (BCF, typically C_plant_/C_medium_) for overall enrichment from the exposure medium, and the translocation factor (TF = C_shoot_/C_root_, unitless) for in-plant mobility. These metrics help distinguish controls on entry into plants from controls on redistribution among tissues. Figure 3 summarizes the major uptake, translocation, accumulation, and metabolism mechanisms of pesticides and antibiotics in plants under agricultural exposure scenarios. Moreover, prior to root uptake, only the dissolved and desorbable fractions in the soil pore water are directly available to plants. It can thus be concluded that physicochemical properties exert a significant influence on the retention of contaminants in the soil, in addition to regulating the actual amount of contaminants that reach the root surface. This distinction is significant because the total amount retained in the soil does not necessarily correspond to the concentration available to plants or the amount accumulated in the final edible tissues. The concentration in soil pore water is often a better indicator of plant exposure than the total residue in bulk soil [161]. This is because pH, organic matter, clay minerals, and coexisting ions jointly control sorption, desorption, and resupply to the root interface [162]. Recent work also showed that dynamic bioavailability measured by DGT can correlate well with antibiotic concentrations in roots and shoots [161].

### 4.1. Migration and Accumulation Mechanisms of Pesticides

The migration and accumulation of pesticides from soil into crops involve vertical transport in soil, root uptake, and subsequent in-plant translocation and sequestration [163]. This trajectory is governed by pesticide physicochemical properties (log Kow, water solubility, Koc, molecular weight) [164], plant anatomical and transpiration traits, and cultivation and water-management practices [165]. Field and laboratory studies now delineate stage-specific controls along this pathway, informing assessments of pesticide fate and food-contamination risks.

To illustrate stage specificity, several studies conceptualize pesticide behavior as three sequential phases: downward movement in soil, root uptake, and in-plant translocation. Representative compounds show contrasting behaviors. Chlorantraniliprole exhibits moderate sorption and can reach stems via both water-driven and sorption-mediated transport, whereas its metabolite IN-EQW78 binds strongly to soil and is far less mobile. By contrast, metolachlor rapidly forms highly soluble metabolites (OA and ESA) that are readily transported with water and accumulate within plant tissues [166]. Crop-specific evidence supports distinct controls on uptake versus redistribution. In maize exposed to imidacloprid, acetamiprid, tricyclazole, azoxystrobin, tebuconazole, and difenoconazole, tissue accumulation was negatively related to Koc and positively related to pore-water concentrations, log Kow, and molecular weight, but negatively related to water solubility. Notably, these relationships reversed when considering TFs, indicating that physicochemical properties can promote entry into roots while simultaneously constraining or facilitating subsequent translocation within plants [167]. Pesticide uptake by roots is not only a passive diffusion process. Recent evidence showed that neonicotinoids mainly enter plant cells through the transmembrane symplastic pathway [168]. The aquaporins PIP1;1 and PIP2;1 can further promote their uptake and accumulation in plant tissues [168].

Compounds also differ in the directionality and magnitude of translocation. Phenamacril in rice and wheat showed strong upward mobility, with TF values up to 6.9, and preferential accumulation in shoots. A small fraction underwent phloem-mediated reverse transport to roots and was exuded to the rhizosphere, where multiple metabolites were detected, indicating that plants can also release transformed residues back to the environment [169]. In-plant redistribution should be evaluated together with biotransformation [R4, R5]. In tomato, cyantraniliprole remained the main residue, but the metabolite profile differed among leaves, flowers, and fruits [170]. Phenamacril can also move through the phloem and then undergo extensive transformation in plant tissues [169].

Across diverse crops including rice [171], wheat [172], cowpea [173], chili pepper [174] pepper, and cucumber, Chinese chive [175] and lettuce [176], pesticide compounds with intermediate log Kow generally show a higher likelihood of both uptake and in-plant redistribution, whereas very hydrophilic or very hydrophobic compounds are more often retained in soil or encounter anatomical barriers to movement. Crop species, growth stage, irrigation regimes, and application methods further modulate net migration.

Notably, some studies have developed dynamic simulation models to characterize the migration of pesticides within plant systems. One such model incorporated key parameters such as the “fruit development window” and “phloem flow dynamics” to successfully simulate the temporal variations in pesticide concentrations in chili pepper and tomato fruits [177]. This modeling approach not only enhances our understanding of pesticide fate and transport mechanisms but also provides a theoretical foundation for designing more targeted and environmentally friendly pesticide application strategies.

### 4.2. Migration and Accumulation Mechanisms of Antibiotics

The uptake of antibiotics by vegetable crops through wastewater irrigation or manure application represents a significant pathway for antibiotic exposure in the food chain, posing potential threats to human health. Therefore, elucidating the mechanisms underlying their migration and accumulation in plants is of critical importance. For antibiotics, total soil concentration is also an incomplete predictor of plant uptake [161,162]. Their uptake is highly sensitive to soil–solution chemistry because most antibiotics are ionizable under environmental conditions [162]. As a result, soils with similar total residues may still differ in plant exposure potential [162].

When antibiotics such as oxytetracycline are introduced into the soil through irrigation or fertilization at environmentally relevant concentrations, they undergo migration and degradation within the soil profile. While soil processes can transiently elevate ARGs and reshape microbiomes [178], their role here is to modulate the antibiotic concentration and speciation at the plant interface, which in turn constrains uptake and translocation.

Studies have demonstrated that different classes of antibiotics exhibit significantly varying accumulation capacities in crops, largely influenced by their physicochemical properties. For instance, in a hydroponic experiment using pak choi, fluoroquinolones showed a markedly higher bioaccumulation potential compared to tetracyclines and sulfonamides. The highest observed bioconcentration factor (BCF) for fluoroquinolones reached up to 0.078, and the accumulation capacity increased with rising antibiotic concentrations in the solution [179,180]. In studies on erythromycin (ERY), the compound exhibited preferential retention in roots, with translocation factors (TFs) less than 1 in both pak choi and water spinach, indicating limited upward mobility [180]. In contrast, fluoroquinolones such as enrofloxacin have been found to accumulate in the leaves and seeds of crops like soybean, common bean, and maize, suggesting a strong capacity for rhizosphere uptake and internal translocation [16]. Antibiotic accumulation is strongly class-dependent [181]. A recent meta-analysis found higher transfer of tetracyclines to leaves than sulfonamides [181]. It also showed that translocation patterns within plants differed among antibiotic classes [181]. Some antibiotics are not only passively absorbed by plants [R8]. Kasugamycin showed transporter-related uptake and phloem redistribution in castor bean, and this process was affected by pH, temperature, and metabolic inhibitors [182].

Antibiotic uptake is compound- and plant-dependent, involving distinct entry routes and vascular transport modes. For example, fluoroquinolones in leafy vegetables have been linked to energy-dependent and/or aquaporin-associated uptake with predominantly xylem-driven translocation [179], whereas kasugamycin shows transporter involvement consistent with active uptake and phloem-associated redistribution that is sensitive to temperature, pH and metabolic inhibitors [182].

Plant surface traits and in planta biotransformation can decouple external retention from internal redistribution of antibiotics. Evidence suggests that root-surface mineral/plaque layers may enhance retention of tetracycline-class compounds while restricting translocation [183], while sulfonamide-class antibiotics can undergo rapid uptake followed by reversible conjugation/deconjugation and detoxification-associated transformations (e.g., CYP450s and glycosyltransferases), with potential downstream effects on primary metabolism [184]. Plant metabolism can further reshape antibiotic residues after uptake [184]. In rice, sulfamethoxazole and N4-acetyl-sulfamethoxazole were rapidly taken up by roots and transported upward [184]. Multiple transformation products were also detected during this process [184].

Overall, antibiotic accumulation in crops is highly context dependent, reflecting chemical properties, plant traits, exposure pathways and metabolic capacity. Integrating these dimensions is necessary to interpret residue patterns and to inform food-safety management and pollution-control strategies.

## 5. Conclusions and Outlook

This review integrates current knowledge on the sources, cross-media transport, and plant uptake of pesticides, antibiotics, and antibiotic resistance genes (ARGs) in agricultural systems. Overall, the available evidence indicates that environmental occurrence alone is not sufficient to predict crop contamination. Rather, the transfer of contaminants from soils, irrigation water, wastewater inputs, and atmospheric deposition into plant tissues is jointly regulated by compound-specific physicochemical properties, environmental conditions, rhizosphere processes, and crop traits. Therefore, cross-media transport and plant uptake should be understood not as isolated topics, but as mechanistically connected stages along a source-to-plant continuum.

Current studies have clearly demonstrated that the environmental sources of these contaminants are diverse, yet their dominant entry pathways differ. For pesticides, direct application, misuse, and overuse remain primary drivers of environmental loading. For antibiotics and ARGs, manure amendment, wastewater discharge, reclaimed-water irrigation, and biosolid application represent major introduction routes. Once released, these contaminants may persist in soils and aquatic systems, undergo redistribution during irrigation and precipitation events, and move across environmental compartments including soil, water, sediment, air, and biota. However, the presence of residues in environmental matrices does not necessarily translate directly into accumulation in edible tissues, because actual plant exposure and uptake depend strongly on bioavailability, speciation, transformation, and plant-specific barriers to entry and translocation.

Despite substantial progress, several important knowledge gaps remain. First, existing studies are much stronger in documenting occurrence patterns than in resolving the mechanistic links between environmental exposure and crop accumulation. Second, current research has focused predominantly on root-mediated uptake, whereas direct foliar deposition, surficial retention, penetration through aboveground tissues, and their relative contributions to internal accumulation remain insufficiently addressed. Third, most monitoring programs emphasize parent compounds, while transformation products and metabolites are still underrepresented, even though they may differ markedly in persistence, mobility, and biological effects. Fourth, ARG dynamics cannot always be inferred directly from measurable antibiotic residues, because ARG persistence may be maintained by microbial hosts, mobile genetic elements, and co-selection pressures even in the absence of detectable antibiotic concentrations. In addition, differences in sampling design, analytical methodology, target compounds, plant species, environmental matrices, and reporting metrics continue to limit cross-study comparability and quantitative synthesis.

Future research should therefore prioritize several directions. First, standardized multi-matrix monitoring frameworks are needed to connect source inputs, environmental transport, and edible-tissue residues across soils, irrigation waters, atmospheric deposition, and crop compartments. Second, more mechanism-oriented studies are required to clarify how root exudates, rhizosphere microbial degradation, sorption–desorption dynamics, plant physiology, and species-specific traits shape contaminant bioavailability, uptake, and translocation. Third, future assessments should move beyond parent compounds to explicitly include transformation products, mixture effects, and plant detoxification pathways. Fourth, predictive models should be developed to link environmental exposure, contaminant fate, plant accumulation, and context-dependent food-safety implications under realistic agricultural scenarios. Climate change should also be considered in future assessments, as shifts in temperature, precipitation, and hydrological extremes may alter contaminant mobility, redistribution, and transformation in agricultural environments. These changes may in turn modify runoff, leaching, and plant exposure patterns under realistic field conditions. Finally, effective mitigation will require not only source control and improved chemical management, but also more specific agricultural measures targeting the environmental dissemination of antibiotic resistance. From a management perspective, reducing dissemination at the source remains critical, and manure pretreatment—especially well-managed composting—has been repeatedly highlighted as a practical route for lowering ARG burdens before land application. Where reclaimed or wastewater-derived irrigation sources are used, mitigation should further rely on fit-for-purpose water treatment and multi-barrier management, rather than on irrigation reuse alone. More broadly, on-farm monitoring, tighter veterinary-drug regulation, and clearer standards for organic fertilizers may serve as complementary measures to reduce environmental ARG spread in agricultural systems. Advancing this field will depend on closer integration of environmental chemistry, plant physiology, agronomy, and microbial ecology, so that contaminant monitoring can be translated into more robust management and regulatory practices.

Advancing this field will depend on closer integration of environmental chemistry, plant physiology, agronomy, and microbial ecology, so that contaminant monitoring can be translated into more robust management and regulatory practices.

## Data Availability

No new data were created or analyzed in this study. Data sharing is not applicable to this article.

## Supplementary Materials

The following supporting information can be downloaded at: https://www.mdpi.com/article/10.3390/foods15081436/s1, Table S1: Key physicochemical properties and policy-related information of the major pesticides discussed in this review, Table S2: Key physicochemical properties and policy-related information of the major antibiotics discussed in this review, Figure S1: Flow diagram of the literature search and selection process.

## References

[1] Pan Y., Ren Y., Luning P.A. (2021). Factors influencing Chinese farmers’ proper pesticide application in agricultural products—A review. Food Control.

[2] Zhang C., Guanming S., Shen J., Hu R.-F. (2015). Productivity effect and overuse of pesticide in crop production in China. J. Integr. Agric..

[3] Shao Y., Wang Y., Yuan Y., Xie Y. (2021). A systematic review on antibiotics misuse in livestock and aquaculture and regulation implications in China. Sci. Total Environ..

[4] Virk A.L., Shakoor A., Abdullah A., Chang S.X., Cai Y. (2024). Pesticide effects on crop physiology, production and soil biological functions. Adv. Agron..

[5] Roskam J.L., Oude Lansink A., Saatkamp H.W. (2023). The Economic Value of Antimicrobial Use in Livestock Production. Antibiotics.

[6] Batuman O., Britt-Ugartemendia K., Kunwar S., Yilmaz S., Fessler L., Redondo A., Chumachenko K., Chakravarty S., Wade T. (2024). The Use and Impact of Antibiotics in Plant Agriculture: A Review. Phytopathology.

[7] Acosta A., Tirkaso W., Nicolli F., Van Boeckel T.P., Cinardi G., Song J. (2025). The future of antibiotic use in livestock. Nat. Commun..

[8] Chen L., Qian Y., Jia Q., Weng R., Zhang X., Li Y., Qiu J. (2023). A national-scale distribution of organochlorine pesticides (OCPs) in cropland soils and major types of food crops in China: Co-occurrence and associated risks. Sci. Total Environ..

[9] Wu J., Wang J., Li Z., Guo S., Li K., Xu P., Ok Y.S., Jones D.L., Zou J. (2022). Antibiotics and antibiotic resistance genes in agri-cultural soils: A systematic analysis. Crit. Rev. Environ. Sci. Technol..

[10] Li Y., Liu C., Li Q., Mao S. (2024). Fluorescence analysis of antibiotics and antibiotic-resistance genes in the environment: A mini review. Chin. Chem. Lett..

[11] Lyu J., Yang L., Zhang L., Ye B., Wang L. (2020). Antibiotics in soil and water in China-a systematic review and source analysis. Environ. Pollut..

[12] Sun J., Pan L., Tsang D.C.W., Zhan Y., Zhu L., Li X. (2018). Organic contamination and remediation in the agricultural soils of China: A critical review. Sci. Total Environ..

[13] Wilkinson J.L., Boxall A.B.A., Kolpin D.W., Leung K.M.Y., Lai R.W.S., Galban-Malagon C., Adell A.D., Mondon J., Metian M., Marchant R.A. (2022). Pharmaceutical pollution of the world’s rivers. Proc. Natl. Acad. Sci. USA.

[14] Chormare R., Kumar M.A. (2022). Environmental health and risk assessment metrics with special mention to biotransfer, bioaccu-mulation and biomagnification of environmental pollutants. Chemosphere.

[15] Gudda F., Odinga E.S., Tang L., Waigi M.G., Wang J., Abdalmegeed D., Gao Y. (2023). Tetracyclines uptake from irrigation water by vegetables: Accumulation and antimicrobial resistance risks. Environ. Pollut..

[16] Marques R.Z., Wistuba N., Brito J.C.M., Bernardoni V., Rocha D.C., Gomes M.P. (2021). Crop irrigation (soybean, bean, and corn) with enrofloxacin-contaminated water leads to yield reductions and antibiotic accumulation. Ecotoxicol. Environ. Saf..

[17] Cui K., Guan S., Liang J., Fang L., Ding R., Wang J., Li T., Dong Z., Wu X., Zheng Y. (2023). Dissipation, metabolism, accumu-lation, processing and risk assessment of fluxapyroxad in cucumber and cowpea vegetables from field to table. Food Chem..

[18] Baudry J., Rebouillat P., Samieri C., Berlivet J., Kesse-Guyot E. (2023). Dietary pesticide exposure and non-communicable diseases and mortality: A systematic review of prospective studies among adults. Environ. Health.

[19] Bacanli M.G. (2024). The two faces of antibiotics: An overview of the effects of antibiotic residues in foodstuffs. Arch. Toxicol..

[20] Shahid A., Ali M.A., Muzammil S., Aslam B., Shahid M., Saqalein M., Akash M.S.H., Almatroudi A., Allemailem K.S., Khurshid M. (2021). Antibiotic Residues in Food Chains; Impact on the Environment and Human Health: A Review. Appl. Ecol. Environ. Res..

[21] Benjamin S., Masai E., Kamimura N., Takahashi K., Anderson R.C., Faisal P.A. (2017). Phthalates impact human health: Epide-miological evidences and plausible mechanism of action. J. Hazard. Mater..

[22] Łozowicka B., Kaczyński P., Wołejko E., Jankowska M., Iwaniuk P., Hrynko I., Rutkowska E., Łuniewski S., Ilyasova G., Jabłońska-Trypuć A. (2025). Comprehensive toxicological multi-year study on pesticides in apples: Control, trends and dietary risk assessment. Food Chem..

[23] Rutkowska E., Kaczyński P., Iwaniuk P., Łozowicka B., Hrynko I., Jankowska M., Konecki R., Rogowska W., Rusiłowska J., Pietkun M. (2025). An extensive pesticide residue study in minor polish vegetables based on critical consumer diets. Food Control.

[24] Chen Q.L., Cui H.L., Su J.Q., Penuelas J., Zhu Y.G. (2019). Antibiotic Resistomes in Plant Microbiomes. Trends Plant Sci..

[25] Zhou Z., Chen H. (2024). Evaluating human exposure to antibiotic resistance genes. Biosaf. Health.

[26] B1 Definitions for the Purposes of the Codex Alimentarius. https://www.fao.org/fao-who-codexalimentarius/codex-texts/procedural-manual/sections/section1/section1-4/en/.

[27] EFSA (2025). The 2023 European Union Report on Pesticide Residues in Food. EFSA J..

[28] PDP Databases and Annual Summaries. https://www.ams.usda.gov/datasets/pdp/pdpdata.

[29] One Health. https://www.who.int/health-topics/one-health.

[30] Delpy L., Astbury C.C., Aenishaenslin C., Ruckert A., Penney T.L., Wiktorowicz M., Ciss M., Benko R., Bordier M. (2024). Integrated surveillance systems for antibiotic resistance in a One Health context: A scoping review. BMC Public Health.

[31] Ruckert A., Harris F., Aenishaenslin C., Aguiar R., Boudreau-LeBlanc A., Carmo L.P., Labonté R., Lambraki I., Parmley E.J., Wiktorowicz M.E. (2024). One Health governance principles for AMR surveillance: A scoping review and conceptual framework. Res. Dir. One Health.

[32] Woolhouse M.E.J. (2024). One Health approaches to tackling antimicrobial resistance. Sci. One Health.

[33] Zaynab M., Fatima M., Sharif Y., Sughra K., Sajid M., Khan K.A., Sneharani A.H., Li S. (2021). Health and environmental effects of silent killers Organochlorine pesticides and polychlorinated biphenyl. J. King Saud. Univ. Sci..

[34] Haddad M.F., Abdullah B.A., AlObeidi H.A.A., Saadi A.M., Haddad M.F. (2024). Antibiotic classification, mechanisms, and in-dications: A review. Int. J. Med. All Body Health Res..

[35] Kosenko Y., Bilous S., Ostapiv N., Zaruma L. (2021). Use of tetracyclines and sulfonamides for the treatment of infectious diseases in animals. Sci. Biol. Sci..

[36] Du J., Liu Q., Pan Y., Xu S., Li H., Tang J. (2023). The Research Status, Potential Hazards and Toxicological Mechanisms of Fluo-roquinolone Antibiotics in the Environment. Antibiotics.

[37] Verhaegen M., Mahillon J., Caulier S., Mingeot-Leclercq M.P., Bragard C. (2024). Data collection on antibiotics for control of plant pathogenic bacteria. EFSA Support. Publ..

[38] Konthonbut P., Kongtip P., Nankongnab N., Tipayamongkholgul M., Yoosook W., Woskie S. (2020). Paraquat exposure of back-pack sprayers in agricultural area in Thailand. Hum. Ecol. Risk Assess..

[39] Mackay J.E., Bernhardt L.T., Smith R.G., Ernakovich J.G. (2023). Tillage and pesticide seed treatments have distinct effects on soil microbial diversity and function. Soil Biol. Biochem..

[40] Chen L., Zhang J., Xia X., Yang Z., Wang B., Long C. (2023). The potential capability of substituting chemical fertilizers with crop straw and human-livestock-poultry manure in areas with different topographic characteristics. Heliyon.

[41] Bochalya M.S., Kumar A., Gandhi V., Kumar R., Chauhan R., Kumar A., Jain A., Madaan S., Saini S., Lalita (2024). Field Efficacy of Fungicides for the Management of Rice Neck Blast (Caused by Pyricularia oryzae) in the North-Western Region of Haryana, India. J. Phytopathol..

[42] Hopkins S.C., Crow W.D., Gore J., Permenter S.T., Sorenson C. (2023). Efficacy of foliar insecticides on thrips in cotton, 2022. Arthropod Manag. Tests.

[43] DeVries T.A., Wright R.J., Crow W. (2024). Evaluation of selected foliar insecticides against western bean cutworm in field corn, 2023. Arthropod Manag. Tests.

[44] Perier J.D., Gruver C.L., Riley D.G., Abney M. (2024). Foliar insecticide treatments in tomato for lepidoptera larvae control, 2022. Arthropod Manag. Tests.

[45] Liu Y., Dong L., Gao Y., Wang K., Meng L., Li B., Zhang D., Liu F. (2025). Improving the efficacy against cucumber root-knot nematode and Alleviating the phytotoxicity of fluopyram by optimizing the application method. Adv. Agrochem..

[46] Mao L., Shi H., Sial M.U., Guo R., Zhang L., Zhang Y., Zhu L., Wu C., Liu X. (2025). Combined use of 1,3-dichloropropene with dimethyl disulfide and chloropicrin for managing potato powdery scab and weed. Sci. Rep..

[47] Onwuchekwa-Henry C.B., Van Ogtrop F., Roche R., Tan D.K.Y. (2023). Evaluation of pre-emergence herbicides for weed man-agement and rice yield in direct-seeded rice in Cambodian lowland ecosystems. Farming Syst..

[48] Kandel Y.R., Lawson M.N., Brown M.T., Chilvers M.I., Kleczewski N.M., Telenko D.E.P., Tenuta A.U., Smith D.L., Mueller D.S. (2023). Field and Greenhouse Assessment of Seed Treatment Fungicides for Management of Sudden Death Syndrome and Yield Response of Soybean. Plant Dis..

[49] Brown M.T., Mueller D.S., Kandel Y.R., Telenko D.E.P. (2023). Influence of Integrated Management Strategies on Soybean Sudden Death Syndrome (SDS) Root Infection, Foliar Symptoms, Yield and Net Returns. Pathogens.

[50] Jayaweera D.P., Ray R.V. (2023). Yield Loss and Integrated Disease Control of Rhizoctonia solani AG2-1 Using Seed Treatment and Sowing Rate of Oilseed Rape. Plant Dis..

[51] Ismail S.M. (2024). Field Efficacy of Seed Treatment Recent Insecticides in Maize on Fall Armyworm (Spodoptera frugiperda, J.E. Smith) and Crop Performance. J. Agric. Sci. Eng..

[52] Song J.H., Wang Y.F., Yin W.X., Huang J.B., Luo C.X. (2021). Effect of Chemical Seed Treatment on Rice False Smut Control in Field. Plant Dis..

[53] Taylor P., Reeder R. (2020). Antibiotic use on crops in low and middle-income countries based on recommendations made by agricultural advisors. CABI Agric. Biosci..

[54] Wakil W., Boukouvala M.C., Kavallieratos N.G., Riasat T., Ghazanfar M.U., Avery P.B. (2024). Acaricidal Efficacy of Abamectin against Tetranychus urticae Populations When Combined with Entomopathogenic Fungi. Horticulturae.

[55] Yu H., Wu M., Li S., Li J., Zou X., Guo Z., Wu Q., Zhang Y., Kong X., Xie W. (2024). A Maximum Dose Bioassay to Assess Efficacy of Spinetoram against Cowpea Thrip Megalurothrips usitatus in China. Insects.

[56] Zhu J., Cai L., Wang Y., Jiang W., Liu X., Wang J., Tian Y., Hu B., Zhao Y. (2025). Evaluation of the new antimicrobial benzio-thiazolinone for management of fire blight disease of pear. BMC Microbiol..

[57] Wallis A., Gu G., Ramachandran P., Reed E., Ottesen A., Nou X., Cox K.D. (2021). Endophytic Bacterial Communities in Apple Leaves Are Minimally Impacted by Streptomycin Use for Fire Blight Management. Phytobiomes J..

[58] Ge L., Zhou Z., Sun K., Huang B., Stanley D., Song Q.S. (2020). The antibiotic jinggangmycin increases brown planthopper (BPH) fecundity by enhancing rice plant sugar concentrations and BPH insulin-like signaling. Chemosphere.

[59] Archer L., Kunwar S., Alferez F., Batuman O., Albrecht U. (2023). Trunk Injection of Oxytetracycline for Huanglongbing Man-agement in Mature Grapefruit and Sweet Orange Trees. Phytopathology.

[60] Deedat F.Z., Faya A.M., Gumbi B.P., Johnston D.M.G., Karpoormath R., Essack S.Y. (2025). The association between antibiotic use in hospitals and residual antibiotic concentrations in hospital effluents: A pilot study. JAC-Antimicrob. Resist..

[61] He D., Li J., Yu W., Zhang Y., Wang B., Wang T., Yang H., Zhang Y., Chen W., Li Y. (2024). Deciphering the removal of antibiotics and the antibiotic resistome from typical hospital wastewater treatment systems. Sci. Total Environ..

[62] Nabgan W., Saeed M., Jalil A.A., Nabgan B., Gambo Y., Ali M.W., Ikram M., Fauzi A.A., Owgi A.H.K., Hussain I. (2022). A state of the art review on electrochemical technique for the remediation of pharmaceuticals containing wastewater. Environ. Res..

[63] Akhil D., Lakshmi D., Senthil Kumar P., Vo D.-V.N., Kartik A. (2021). Occurrence and removal of antibiotics from industrial wastewater. Environ. Chem. Lett..

[64] Dong Z., Hu J., Wang P., Han G., Jia Z. (2024). Antibiotics in Wastewater Treatment Plants in Tangshan: Perspectives on Temporal Variation, Residents’ Use and Ecological Risk Assessment. Water.

[65] Li Y., Wang J., Lin C., Lian M., He M., Liu X., Ouyang W. (2024). Occurrence, removal efficiency, and emission of antibiotics in the sewage treatment plants of a low-urbanized basin in China and their impact on the receiving water. Sci. Total Environ..

[66] Zeng H., Li J., Zhao W., Xu J., Xu H., Li D., Zhang J. (2022). The Current Status and Prevention of Antibiotic Pollution in Groundwater in China. Int. J. Environ. Res. Public Health.

[67] Heyde B.J., Braun M., Soufi L., Lüneberg K., Gallego S., Amelung W., Axtmann K., Bierbaum G., Glaeser S.P., Grohmann E. (2025). Transition from irrigation with untreated wastewater to treated wastewater and associated benefits and risks. npj Clean. Water.

[68] Nahim-Granados S., Quon H., Polo-Lopez M.I., Oller I., Aguera A., Jiang S. (2024). Assessment of antibiotic-resistant infection risks associated with reclaimed wastewater irrigation in intensive tomato cultivation. Water Res..

[69] Harrison J.C., Morgan G.V., Kuppravalli A., Novak N., Farrell M., Bircher S., Garner E., Ashbolt N.J., Pruden A., Muenich R.L. (2024). Determinants of antimicrobial resistance in biosolids: A systematic review, database, and meta-analysis. Sci. Total Environ..

[70] Paiano R.B., Morrison E.I., LeBlanc S.J. (2024). Randomized clinical trial of ketoprofen or ceftiofur for treatment of metritis in dairy cows. J. Dairy Sci..

[71] Li Y., Du J., Yang Q., Li R., Jin S., Guo X., Wang X., Zhang W., Xu L. (2024). The withdrawal time of enrofloxacin, sulfachloro-pyrazine sodium, and doxycycline as well as the in vitro binding interaction with melanin in black-feathered silky fowl. Food Chem. X.

[72] Abate T.A., Birhanu A.G. (2025). Antibiotic Use in Livestock and Environmental Antibiotic Resistance: A Narrative Review. Environ. Health Insights.

[73] Zha Y., Li Q., Liu H., Ge Y., Wei Y., Wang H., Zhang L., Fan J., Chen Y., Zhang C. (2023). Occurrence and ecological risk assessment of antibiotics in manure and the surrounding soil from typical chicken farms in Hangzhou, China. Front. Environ. Sci..

[74] Frey L., Tanunchai B., Glaser B. (2022). Antibiotics residues in pig slurry and manure and its environmental contamination potential. A meta-analysis. Agron. Sustain. Dev..

[75] Huygens J., Daeseleire E., Mahillon J., Van Elst D., Decrop J., Meirlaen J., Dewulf J., Heyndrickx M., Rasschaert G. (2021). Presence of Antibiotic Residues and Antibiotic Resistant Bacteria in Cattle Manure Intended for Fertilization of Agricultural Fields: A One Health Perspective. Antibiotics.

[76] Gross A., Glaser B. (2021). Meta-analysis on how manure application changes soil organic carbon storage. Sci. Rep..

[77] Dong Y., Das S., Parsons J.R., Praetorius A., de Rijke E., Helmus R., Slootweg J.C., Jansen B. (2023). Simultaneous detection of pesticides and pharmaceuticals in three types of bio-based fertilizers by an improved QuEChERS method coupled with UHPLC-q-ToF-MS/MS. J. Hazard. Mater..

[78] Batool F., Hussain M.I., Nazar S., Bashir H., Khan Z.I., Ahmad K., Alnuwaiser M.A., Yang H.-H. (2023). Potential of sewage irrigation for heavy metal contamination in soil–wheat grain system: Ecological risk and environmental fate. Agric. Water Manag..

[79] Fengle Y., Xianzhi Z., Jinhua L., Hongfeng Z., Fangming J., Zhou B. (2021). Analysis and evaluation of the treatment and reuse of tailwater: A case study in Erhai Lake. J. Clean. Prod..

[80] Wang L., Zhang Z.F., Liu L.Y., Zhu F.J., Ma W.L. (2023). National-scale monitoring of historic used organochlorine pesticides (OCPs) and current used pesticides (CUPs) in Chinese surface soil: Old topic and new story. J. Hazard. Mater..

[81] Tao Y., Jia C., Jing J., Zhang J., Yu P., He M., Wu J., Chen L., Zhao E. (2021). Occurrence and dietary risk assessment of 37 pes-ticides in wheat fields in the suburbs of Beijing, China. Food Chem..

[82] Chen W., Zeng F., Liu W., Bu J., Hu G., Xie S., Yao H., Zhou H., Qi S., Huang H. (2021). Organochlorine Pesticides in Karst Soil: Levels, Distribution, and Source Diagnosis. Int. J. Environ. Res. Public Health.

[83] Rodriguez-Seijo A., Perez-Rodriguez P., Arias-Estevez M., Gomez-Armesto A., Conde-Cid M., Santas-Miguel V., Campil-lo-Cora C., Ollio I., Lloret E., Martinez-Martinez S. (2025). Occurrence, persistence and risk assessment of pesticide residues in European wheat fields: A continental scale approach. J. Hazard. Mater..

[84] Knuth D., Gai L., Silva V., Harkes P., Hofman J., Sudoma M., Bilkova Z., Alaoui A., Mandrioli D., Paskovic I. (2024). Pesticide Residues in Organic and Conventional Agricultural Soils across Europe: Measured and Predicted Concentrations. Environ. Sci. Technol..

[85] Froger C., Jolivet C., Budzinski H., Pierdet M., Caria G., Saby N.P.A., Arrouays D., Bispo A. (2023). Pesticide Residues in French Soils: Occurrence, Risks, and Persistence. Environ. Sci. Technol..

[86] Navarro I., de la Torre A., Sanz P., Abrantes N., Campos I., Alaoui A., Christ F., Alcon F., Contreras J., Glavan M. (2024). Assessing pesticide residues occurrence and risks in water systems: A Pan-European and Argentina perspective. Water Res..

[87] Barbieri M.V., Peris A., Postigo C., Moya-Garces A., Monllor-Alcaraz L.S., Rambla-Alegre M., Eljarrat E., Lopez de Alda M. (2021). Evaluation of the occurrence and fate of pesticides in a typical Mediterranean delta ecosystem (Ebro River Delta) and risk as-sessment for aquatic organisms. Environ. Pollut..

[88] Zhang R., Du J., Dong X., Huang Y., Xie H., Chen J., Li X., Kadokami K. (2021). Occurrence and ecological risks of 156 phar-maceuticals and 296 pesticides in seawater from mariculture areas of Northeast China. Sci. Total Environ..

[89] Li H., Jiang W., Pan Y., Li F., Wang C., Tian H. (2021). Occurrence and partition of organochlorine pesticides (OCPs) in water, sediment, and organisms from the eastern sea area of Shandong Peninsula, Yellow Sea, China. Mar. Pollut. Bull..

[90] Zhang Y., Qin P., Lu S., Liu X., Zhai J., Xu J., Wang Y., Zhang G., Liu X., Wan Z. (2021). Occurrence and risk evaluation of organophosphorus pesticides in typical water bodies of Beijing, China. Environ. Sci. Pollut. Res. Int..

[91] Dugan S.T., Muhammetoglu A., Uslu A. (2023). A combined approach for the estimation of groundwater leaching potential and environmental impacts of pesticides for agricultural lands. Sci. Total Environ..

[92] Xie Q., Tan J., Li H., Xu X., Huang G., Huo Z. (2025). Assessment of the environmental health and human exposure risk of emerging contaminants in groundwater of a typical agricultural irrigation area in the North China Plain. J. Hazard. Mater..

[93] Broers H.P., Kivits T., Sultenfuss J., Ten Harkel M., van Vliet M. (2024). Mobility and persistence of pesticides and emerging con-taminants in age-dated and redox-classified groundwater under a range of land use types. Sci. Total Environ..

[94] Li C., Qi X., Wang Y., Meng Q., Li W., Liu L., Zheng Y., Cui H. (2023). Organochlorine Pesticides in Soil–Groundwater–Plant System in a Famous Agricultural Production Area in China: Spatial Distribution, Source Identification and Migration Prediction. Water.

[95] Rajan S., Parween M., Raju N.J. (2023). Pesticides in the hydrogeo-environment: A review of contaminant prevalence, source and mobilisation in India. Environ. Geochem. Health.

[96] Goedjen G.J., Capel P.D., Barry J.D., Arnold W.A. (2024). Occurrence and distribution of neonicotinoids and fiproles within groundwater in Minnesota: Effects of lithology, land use and geography. Sci. Total Environ..

[97] Park S., Choi H., Kim D.-H., Kim H.-K. (2024). Evaluation of the Health Risk and Distribution Characteristics of Pesticides in Shallow Groundwater, South Korea. Water.

[98] Fang L., Chen C., Li S., Ye P., Shi Y., Sharma G., Sarkar B., Shaheen S.M., Lee S.S., Xiao R. (2023). A comprehensive and global evaluation of residual antibiotics in agricultural soils: Accumulation, potential ecological risks, and attenuation strategies. Ecotoxicol. Environ. Saf..

[99] Gu J., Chen C., Huang X., Mo J., Xie Q., Zeng Q. (2021). Occurrence and risk assessment of tetracycline antibiotics in soils and vegetables from vegetable fields in Pearl River Delta, South China. Sci. Total Environ..

[100] Huang X., Liu C., Li K., Liu F., Liao D., Liu L., Zhu G., Liao J. (2013). Occurrence and distribution of veterinary antibiotics and tetracycline resistance genes in farmland soils around swine feedlots in Fujian Province, China. Environ. Sci. Pollut. Res. Int..

[101] Wei R., Ge F., Zhang L., Hou X., Cao Y., Gong L., Chen M., Wang R., Bao E. (2016). Occurrence of 13 veterinary drugs in animal manure-amended soils in Eastern China. Chemosphere.

[102] Addis T.Z., Adu J.T., Kumarasamy M., Demlie M. (2024). Occurrence of Trace-Level Antibiotics in the Msunduzi River: An Inves-tigation into South African Environmental Pollution. Antibiotics.

[103] Wu S., Hua P., Gui D., Zhang J., Ying G., Krebs P. (2022). Occurrences, transport drivers, and risk assessments of antibiotics in typical oasis surface and groundwater. Water Res..

[104] Apreja M., Sharma A., Balda S., Kataria K., Capalash N., Sharma P. (2022). Antibiotic residues in environment: Antimicrobial resistance development, ecological risks, and bioremediation. Environ. Sci. Pollut. Res. Int..

[105] Gillieatt B.F., Coleman N.V. (2024). Unravelling the mechanisms of antibiotic and heavy metal resistance co-selection in environmental bacteria. FEMS Microbiol Rev..

[106] Klümper U., Fang P., Li B., Xia Y., Frigon D., Hamilton K.A., Quon H., Berendonk T.U., de la Cruz Barron M. (2025). Towards the integration of antibiotic resistance gene mobility into environmental surveillance and risk assessment. npj Antimicrob. Resist..

[107] Xu W., Pan Z., Wu Y., An X.L., Wang W., Adamovich B., Zhu Y.G., Su J.Q., Huang Q. (2024). A database on the abundance of environmental antibiotic resistance genes. Sci. Data.

[108] Zhu C., Wu L., Ning D., Tian R., Gao S., Zhang B., Zhao J., Zhang Y., Xiao N., Wang Y. (2025). Global diversity and distribution of antibiotic resistance genes in human wastewater treatment systems. Nat. Commun..

[109] Zheng D.S., Yin G.Y., Liu M., Hou L.J., Yang Y., Van Boeckel T.P., Zheng Y.L., Li Y. (2022). Global biogeography and projection of soil antibiotic resistance genes. Sci. Adv..

[110] Heeb F., Singer H., Pernet-Coudrier B., Qi W., Liu H., Longree P., Muller B., Berg M. (2012). Organic micropollutants in rivers downstream of the megacity Beijing: Sources and mass fluxes in a large-scale wastewater irrigation system. Environ. Sci. Technol..

[111] Richards L.A., Guo S., Lapworth D.J., White D., Civil W., Wilson G.J.L., Lu C., Kumar A., Ghosh A., Khamis K. (2023). Emerging organic contaminants in the River Ganga and key tributaries in the middle Gangetic Plain, India: Characterization, distribution & controls. Environ. Pollut..

[112] Cheng H., Shen G., Zhi H., Tao S. (2023). Spatiotemporal distribution characteristics and ecological risk of emerging contaminants in a typical river on the Tibetan Plateau. Chin. Sci. Bull..

[113] Pan L., Feng X., Cao M., Zhang S., Huang Y., Xu T., Jing J., Zhang H. (2019). Determination and distribution of pesticides and antibiotics in agricultural soils from northern China. RSC Adv..

[114] Backhaus T., Faust M. (2012). Predictive environmental risk assessment of chemical mixtures: A conceptual framework. Environ. Sci. Technol..

[115] Drakvik E., Altenburger R., Aoki Y., Backhaus T., Bahadori T., Barouki R., Brack W., Cronin M.T.D., Demeneix B., Hougaard Bennekou S. (2020). Statement on advancing the assessment of chemical mixtures and their risks for human health and the environment. Environ. Int..

[116] Escher B., Braun G., Zarfl C. (2020). Exploring the Concepts of Concentration Addition and Independent Action Using a Linear Low-Effect Mixture Model. Environ. Toxicol. Chem..

[117] Cedergreen N., Christensen A.M., Kamper A., Kudsk P., Mathiassen S.K., Streibig J.C., Sørensen H. (2008). A review of independent action compared to concentration addition as reference models for mixtures of compounds with different molecular target sites. Environ. Toxicol. Chem..

[118] Martin O., Scholze M., Ermler S., McPhie J., Bopp S.K., Kienzler A., Parissis N., Kortenkamp A. (2021). Ten years of research on synergisms and antagonisms in chemical mixtures: A systematic review and quantitative reappraisal of mixture studies. Environ. Int..

[119] Cedergreen N. (2014). Quantifying synergy: A systematic review of mixture toxicity studies within environmental toxicology. PLoS ONE.

[120] Finckh S., Beckers L.M., Busch W., Carmona E., Dulio V., Kramer L., Krauss M., Posthuma L., Schulze T., Slootweg J. (2022). A risk based assessment approach for chemical mixtures from wastewater treatment plant effluents. Environ. Int..

[121] Yuan S., Arellano A.F., Knickrehm L., Chang H.I., Castro C.L., Furlong M. (2024). Towards quantifying atmospheric dispersion of pesticide spray drift in Yuma County Arizona. Atmos. Environ..

[122] Perriot B., Pasquier D., Hudebine Y., Verpont F., Verges A., Codis S., Douzals J.P., Bedos C., Grimbuhler S., Sellam M. (2024). Spray drift in field crops: A dataset to analyse the influence of air induction nozzles, hedges, and their combination on the reduction of sedimentary drift, aerial drift and exposure of bystanders. Data Brief.

[123] Butts T.R., Fritz B.K., Kouame K.B., Norsworthy J.K., Barber L.T., Ross W.J., Lorenz G.M., Thrash B.C., Bateman N.R., Adamczyk J.J. (2022). Herbicide spray drift from ground and aerial applications: Implications for potential pollinator foraging sources. Sci. Rep..

[124] Zhang Y., Li Z., Reichenberger S., Gentil-Sergent C., Fantke P. (2024). Quantifying pesticide emissions for drift deposition in com-parative risk and impact assessment. Environ. Pollut..

[125] Brüggemann M., Mayer S., Brown D., Terry A., Rüdiger J., Hoffmann T. (2024). Measuring pesticides in the atmosphere: Current status, emerging trends and future perspectives. Environ. Sci. Eur..

[126] Mayer L., Degrendele C., Senk P., Kohoutek J., Pribylova P., Kukucka P., Melymuk L., Durand A., Ravier S., Alastuey A. (2024). Widespread Pesticide Distribution in the European Atmosphere Questions their Degradability in Air. Environ. Sci. Technol..

[127] Birgul A., Guzel E., Daglioglu N., Tasdemir Y., Cindoruk S.S., Kurt-Karakus P.B. (2025). Evaluation of the concentrations of current use pesticides (CUPs) in urban air and rainfall, and their wet deposition flux in a metropolitan environment. Sci. Total Environ..

[128] Dias M.A., Santos V.S., Vizioli B.C., Ferreira B.S., Montagner C.C. (2025). Pesticides in rainwater: A two-year occurrence study in an unexplored environmental compartment in regions with different land use in the State of Sao Paulo–Brazil. Chemosphere.

[129] Briceño G., Palma G., Schalchli H., Durán P., Llafquén C., Huenchupán A., Rodríguez-Rodríguez C., Diez M.C. (2025). Ad-sorption–Desorption Behaviour of Imidacloprid, Thiamethoxam, and Clothianidin in Different Agricultural Soils. Agriculture.

[130] Cuervo-Pérez A., Del Carmen Díaz-Nava M., Solache-Ríos M., Albiter-López M.V. (2025). Atrazine Sorption Kinetics of Two Mexican Agricultural Soils: Effect of Organic Matter. Soil. Sediment. Contam. An. Int. J..

[131] Mamy L., Marin-Benito J.M., Alletto L., Justes E., Ubertosi M., Munier-Jolain N., Nicolardot B., Bonnet C., Moeys J., Larsbo M. (2024). Measurement and modelling of water flows and pesticide leaching under low input cropping systems. Sci. Total Environ..

[132] Pirlot C., Blondel A., Krings B., Durenne B., Pigeon O., Degre A. (2025). Pesticide fate under varying cropping systems and soil depths: A study using leaching experiments and inverse modelling. J. Contam. Hydrol..

[133] Fišer C., Zagmajster M., Jemec Kokalj A., Mali N., Šumrada T., Glavan M., Hose G.C., Schwartz B., Lorenzo T.D., Griebler C. (2025). Toward sustainable irrigation practices safeguarding groundwater biodiversity and ecosystem services. BioScience.

[134] Cheng X., Hou H., Li R., Zheng C., Liu H. (2020). Adsorption behavior of tetracycline on the soil and molecular insight into the effect of dissolved organic matter on the adsorption. J. Soils Sediments.

[135] Stando K., Korzeniewska E., Felis E., Harnisz M., Buta-Hubeny M., Bajkacz S. (2022). Determination of antimicrobial agents and their transformation products in an agricultural water-soil system modified with manure. Sci. Rep..

[136] Zhang Y., Zhu D., Xie J., Xie J., Yuan C., Shi X. (2025). Vertical migration of antibiotics during rainfall throughout a year in long-term manure-fertilized soils differing in pH. J. Hazard. Mater..

[137] Zhang Q., Li Y., Kroeze C., van de Schans M.G.M., Baartman J., Yang J., Li S., Xu W., Wang M., Ma L. (2025). More inputs of antibiotics into groundwater but less into rivers as a result of manure management in China. Environ. Sci. Ecotechnol..

[138] Zuo R., Liu X., Zhang Q., Wang J., Yang J., Teng Y., Chen X., Zhai Y. (2021). Sulfonamide antibiotics in groundwater and their migration in the vadose zone: A case in a drinking water resource. Ecol. Eng..

[139] Ferrey M.L., Coreen Hamilton M., Backe W.J., Anderson K.E. (2018). Pharmaceuticals and other anthropogenic chemicals in at-mospheric particulates and precipitation. Sci. Total Environ..

[140] Yang Q., Gao Y., Ke J., Show P.L., Ge Y., Liu Y., Guo R., Chen J. (2021). Antibiotics: An overview on the environmental occurrence, toxicity, degradation, and removal methods. Bioengineered.

[141] Snow D.D., Cassada D.A., Biswas S., Malakar A., D’Alessio M., Carter L.J., Johnson R.D., Sallach J.B. (2019). Detection, occur-rence, and fate of emerging contaminants in agricultural environments (2019). Water Environ. Res..

[142] Jiang Y., Zhang Y., Liang Y., Liu W., Wang Y., Yang J., Qiu R., Di H.J., A D. (2024). Migration of nanocolloid-carrying antibiotics in paddy red soil during the organic fertilization process. Sci. Total Environ..

[143] Ellabaan M.M.H., Munck C., Porse A., Imamovic L., Sommer M.O.A. (2021). Forecasting the dissemination of antibiotic resistance genes across bacterial genomes. Nat. Commun..

[144] Michaelis C., Grohmann E. (2023). Horizontal Gene Transfer of Antibiotic Resistance Genes in Biofilms. Antibiotics.

[145] Partridge S.R., Kwong S.M., Firth N., Jensen S.O. (2018). Mobile Genetic Elements Associated with Antimicrobial Resistance. Clin. Microbiol. Rev..

[146] Noel H.R., Petrey J.R., Palmer L.D. (2022). Mobile genetic elements in Acinetobacter antibiotic-resistance acquisition and dissemi-nation. Ann. N. Y Acad. Sci..

[147] Congilosi J.L., Aga D.S. (2021). Review on the fate of antimicrobials, antimicrobial resistance genes, and other micropollutants in manure during enhanced anaerobic digestion and composting. J. Hazard. Mater..

[148] Yue Z., Zhang J., Zhou Z., Ding C., Wan L., Liu J., Chen L., Wang X. (2021). Pollution characteristics of livestock faeces and the key driver of the spread of antibiotic resistance genes. J. Hazard. Mater..

[149] Mu M., Yang F., Han B., Tian X., Zhang K. (2022). Manure application: A trigger for vertical accumulation of antibiotic resistance genes in cropland soils. Ecotoxicol. Environ. Saf..

[150] Li H., Zheng X., Tan L., Shao Z., Cao H., Xu Y. (2022). The vertical migration of antibiotic-resistant genes and pathogens in soil and vegetables after the application of different fertilizers. Environ. Res..

[151] Zhang Y., Zhang Y., Xie J., Yuan C., Zhu D., Shi X. (2024). Vertical migration and leaching behavior of antibiotic resistance genes in soil during rainfall: Impact by long-term fertilization. Water Res..

[152] Dong H., Chen Y., Wang J., Zhang Y., Zhang P., Li X., Zou J., Zhou A. (2021). Interactions of microplastics and antibiotic re-sistance genes and their effects on the aquaculture environments. J. Hazard. Mater..

[153] Zainab S.M., Junaid M., Xu N., Malik R.N. (2020). Antibiotics and antibiotic resistant genes (ARGs) in groundwater: A global review on dissemination, sources, interactions, environmental and human health risks. Water Res..

[154] Li J., Zhou L., Zhang X., Xu C., Dong L., Yao M. (2016). Bioaerosol emissions and detection of airborne antibiotic resistance genes from a wastewater treatment plant. Atmos. Environ..

[155] Gao M., Qiu T., Sun Y., Wang X. (2018). The abundance and diversity of antibiotic resistance genes in the atmospheric environment of composting plants. Environ. Int..

[156] Li L., Wang Q., Bi W., Hou J., Xue Y., Mao D., Das R., Luo Y., Li X. (2020). Municipal Solid Waste Treatment System Increases Ambient Airborne Bacteria and Antibiotic Resistance Genes. Environ. Sci. Technol..

[157] Li J., Cao J., Zhu Y.-g., Chen Q.-l., Shen F., Wu Y., Xu S., Fan H., Da G., Huang R.-J. (2018). Global Survey of Antibiotic Resistance Genes in Air. Environ. Sci. Technol..

[158] Xie J., Jin L., He T., Chen B., Luo X., Feng B., Huang W., Li J., Fu P., Li X. (2019). Bacteria and Antibiotic Resistance Genes (ARGs) in PM(2.5) from China: Implications for Human Exposure. Environ. Sci. Technol..

[159] Caliz J., Subirats J., Triado-Margarit X., Borrego C.M., Casamayor E.O. (2022). Global dispersal and potential sources of antibiotic resistance genes in atmospheric remote depositions. Environ. Int..

[160] Song D., Tang X., Tariq A., Pan K., Li D. (2023). Regional distribution and migration potential of antibiotic resistance genes in croplands of Qinghai Tibet Plateau. Environ. Res..

[161] Song M., Su Y., Jiang L., Peng K., Li J., Liu S., Sun Y., Chen C.E., Luo C. (2023). Assessing the bioavailability of antibiotics in soil with the diffusive gradients in thin films (DGT). J. Hazard. Mater..

[162] Nkoh J.N., Shang C., Okeke E.S., Ejeromedoghene O., Oderinde O., Etafo N.O., Mgbechidinma C.L., Bakare O.C., Meugang E.F. (2024). Antibiotics soil-solution chemistry: A review of environmental behavior and uptake and transformation by plants. J. Environ. Manag..

[163] Kaur H., Kaur R., Singh S., Jagota N., Sharma A. (2024). Pesticide biology in plants: Plant uptake, translocation, and accumulation. Pestic. Environ..

[164] Mendes K.F., Da Silva A. (2022). Applied Weed and Herbicide Science.

[165] Hua Y., Yuan Y., Qin Y., Zhang C., Wang X., Feng S., Lu Y. (2022). Advances in the Agro-Environment Migration of Organic Chemical Pollutants and Their Biotransformation in Crops. Agronomy.

[166] Jung G., Lee H., Choi H., Noh H. (2025). Investigating the three-stage vertical movement of chlorantraniliprole and metolachlor, and their metabolites in soil and rice plants: A long-term lysimeter and soil column study. J. Hazard. Mater..

[167] Wang F., Li X., Yu S., He S., Cao D., Yao S., Fang H., Yu Y. (2021). Chemical factors affecting uptake and translocation of six pesticides in soil by maize (*Zea mays* L.). J. Hazard. Mater..

[168] Wan Q., Li Y., Cheng J., Wang Y., Ge J., Liu T., Ma L., Li Y., Liu J., Zhou C. (2024). Two aquaporins, PIP1;1 and PIP2;1, mediate the uptake of neonicotinoid pesticides in plants. Plant Commun..

[169] Li R., Chang J., Pan X., Dong F., Wang G., Li Z., Zheng Y., Li Y. (2023). Phloem Redistribution of Pesticide Phenamacril in Plants Followed by Extensive Biotransformation. Environ. Sci. Technol. Lett..

[170] Huynh K., Leonard E., Chong J.H., Palmer C., Tharayil N. (2021). Persistence and metabolism of the diamide insecticide cyantraniliprole in tomato plants. Sci. Rep..

[171] Liu J., Cheng J., Zhou C., Ma L., Chen X., Li Y., Sun X., Yan X., Geng R., Wan Q. (2023). Uptake kinetics and subcellular distribution of three classes of typical pesticides in rice plants. Sci. Total Environ..

[172] Liu Q., Liu Y., Dong F., Sallach J.B., Wu X., Liu X., Xu J., Zheng Y., Li Y. (2021). Uptake kinetics and accumulation of pesticides in wheat (*Triticum aestivum* L.): Impact of chemical and plant properties. Environ. Pollut..

[173] Zhang S., Zhang Y., Ren S., Lu H., Li J., Liang X., Wang L., Li Y., Wang M., Zhang C. (2023). Uptake, translocation and me-tabolism of acetamiprid and cyromazine by cowpea (*Vigna unguiculata* L.). Environ. Pollut..

[174] Song S.-S., Yu Q., Yuan L.-W., Anwar W., Li Q., Hao Q., Wu G.-L., Li Y., Lai Y.-S. (2023). Absorption, translocation, and ac-cumulation of the fungicide triadimefon in Pak choi (*Brassica rapa* var chinensis), pepper (*Capsicum annuum*), and cucumber (*Cucumis sativus*). Environ. Monit. Assess..

[175] Wang Y., Li X., Shen J., Lang H., Dong S., Zhang L., Fang H., Yu Y. (2022). Uptake, translocation, and metabolism of thia-methoxam in soil by leek plants. Environ. Res..

[176] Tao Y., Jia C., Jing J., Zhao M., Yu P., He M., Chen L., Zhao E. (2021). Uptake, Translocation, and Biotransformation of Neon-icotinoid Imidaclothiz in Hydroponic Vegetables: Implications for Potential Intake Risks. J. Agric. Food Chem..

[177] Rein A., Trapp S., Fantke P., Yalcin M., Turgut N., Ahat C., Camci E., Turgut C. (2025). Uptake and translocation of pesticides in pepper and tomato plants. Pest. Manag. Sci..

[178] Liu Z., Jin Y., Yu Z., Liu Z., Zhang B., Chi T., Cheng D., Zhu L., Hu B. (2023). Vertical migration and dissipation of oxytetracycline induces the recoverable shift in microbial community and antibiotic resistance. Sci. Total Environ..

[179] Yu X., Chen J., Liu X., Sun Y., He H. (2022). The mechanism of uptake and translocation of antibiotics by pak choi (*Brassica rapa* subsp. chinensis). Sci. Total Environ..

[180] Bao Q., Wang Y., Tang S., Ye F., Yu Z., Ye Q., Wang W. (2022). Uptake and accumulation of erythromycin in leafy vegetables and induced phytotoxicity and dietary risks. Sci. Total Environ..

[181] Nybom I., Bucheli T.D., Garland G. (2024). Antibiotics Uptake from Soil and Translocation in the Plants—Meta-analysis. Chimia.

[182] Zhang H., Zhang C., Xiang X., Zhang Q., Zhao W., Wei G., Hu A. (2022). Uptake and transport of antibiotic kasugamycin in castor bean (*Ricinus communis* L.) seedlings. Front. Microbiol..

[183] Tang J., Wang P., Xie Z., Wang Z., Hu B. (2021). Effect of iron plaque on antibiotic uptake and metabolism in water spinach (*Ip-omoea aquatic Forsk*.) grown in hydroponic culture. J. Hazard. Mater..

[184] Ai T., Yao S., Yu Y., Peng K., Jin L., Zhu X., Zhou H., Huang J., Sun J., Zhu L. (2024). Transformation process and phytotoxicity of sulfamethoxazole and N4-acetyl-sulfamethoxazole in rice. Sci. Total Environ..

