# Supplementary figures and images for: An Integrated Review of Pesticides and Antibiotics in Agricultural Environments: Occurrence, Cross-Media Transport, and Plant Uptake
Source: Foods. 2026 Apr 20;15(8):1436. doi: 10.3390/foods15081436 (PMC13114842; doi:10.3390/foods15081436)

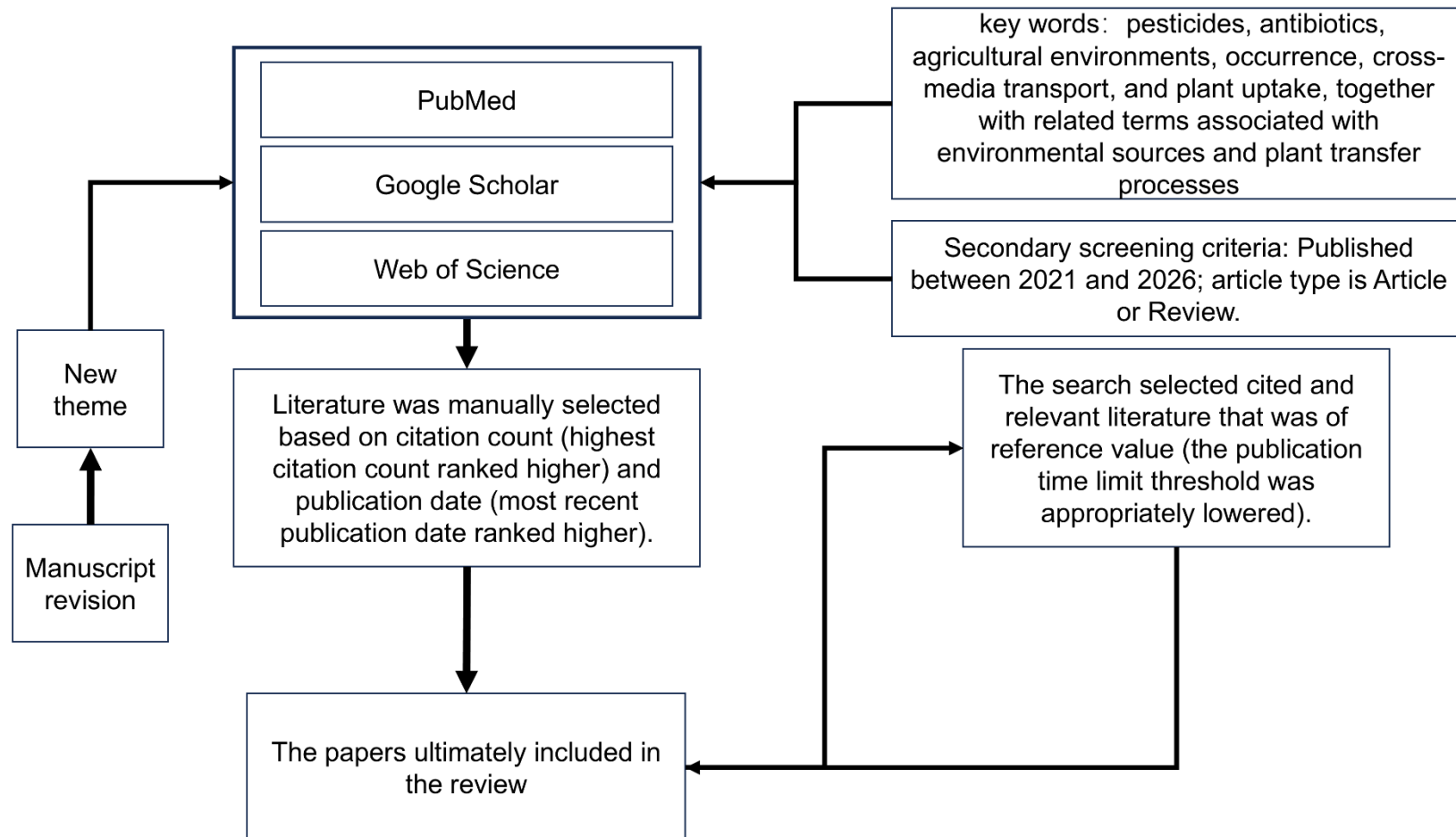

Supplement: Supplementary file 1 [file foods-15-01436-s001.zip › Supplementary materials 1.pdf]
